# Validation of the Turkish Version of the Rapid Eye Movement Sleep Behavior Disorder Questionnaire

**DOI:** 10.1155/2016/8341651

**Published:** 2016-06-01

**Authors:** Itır Tarı Cömert, Zerrin Pelin, Tolga Arıcak, Saadet Yapan

**Affiliations:** ^1^Department of Psychology, Fatih Sultan Mehmet University, Valide-i Atik Mah. Kartalbaba Cadde No. 36, Üsküdar, Istanbul, Turkey; ^2^Department of Health Sciences, Hasan Kalyoncu University, Havaalanı Yolu Üzeri 8 km., Şahinbey, Gaziantep, Turkey; ^3^Somnus Sleep and Neurologic Disorders Clinic, Istanbul, Turkey; ^4^Department of Psychology, Hasan Kalyoncu University, Havaalanı Yolu Üzeri 8 km., Şahinbey, Gaziantep, Turkey

## Abstract

The aim of this study was to assess the validity and reliability of a Turkish version of the rapid eye movement sleep behavior disorder questionnaire (the RBDSQ-T) for identifying patients with rapid eye movement sleep behavior disorder (RBD) and to ensure that this tool can be applied in Turkish language. Three groups were enrolled to validate the RBDSQ-T: 78 healthy controls, 17 patients previously diagnosed with RBD, and 28 patients with obstructive sleep apnea syndrome (OSAS). Based on a cut-off score of five, the RBDSQ-T was able to discriminate RBD patients from healthy controls with sensitivity of 100% and specificity of 87%. Accordingly, 63% of patients were correctly diagnosed using the RBDSQ-T. Similarly, with a cut-off score of five, the RBDSQ-T was able to discriminate RBD from OSAS with sensitivity of 100% and specificity of 64%. Assessment of test-retest reliability and internal consistency reliability using Kuder-Richardson 20 analysis revealed a test-retest correlation coefficient of 0.95 and a Kuder-Richardson 20 value of 0.82. The findings demonstrate that the RBDSQ-T is a valid and reliable tool.

## 1. Introduction

Rapid eye movement sleep behavior disorder (RBD) is characterized by actions during rapid eye movement (REM) sleep, such as talking, shouting, kicking, standing up, and even falling out of bed. These actions occur due to intermittent loss of muscle atonia which is normally associated with REM sleep, and affected patients move in accord with their dream contents [1]. Dream content for RBD patients is usually unpleasant and violent; the disorder is problematic in that patients and their sleeping partners may become injured by such violent and even life-threatening behaviors [2].

According to the International Classification of Sleep Disorders-II (ICSD-II), diagnosis of RBD requires video-polysomnographic evidence of the absence of atonia associated with movements during REM sleep [3]. However, video-polysomnography is not a practical method to screen RBD patients at population scale. An easily applicable and practical method is needed to diagnose this disorder. To address this, Stiasny-Kolster developed a questionnaire for screening RBD, the REM sleep behavior disorder questionnaire (RBDSQ), which was published in English and German versions in 2007 [4]. Subsequently, this instrument was also validated for the Japanese population as a screening tool for RBD [5].

The exact prevalence of RBD has not yet been clearly documented, but studies conducted on different age groups of elderly people in Hong Kong have revealed an estimated range of 0.38% to 0.50% [6, 7]. Another investigation of 70-year-old or older residents of Hong Kong indicated an estimated RBD prevalence of 0.38% based on polysomnographic data [8]. According to data obtained from a 2013 census in Turkey, nearly 6 million people in this country are now older than 65 years of age [9]. Based on an estimated prevalence of 0.38% there are approximately 23,000 cases of RBD in Turkey currently.

Rapid eye movement sleep behavior disorder is recognized as one of the most common causes of sleep-related violence [10]. The potential for injury to oneself or a bed partner raises difficult forensic medicine questions. Violent behavior during sleep is a rare occurrence but one with troubling implications for adjudicating criminal responsibility. Apart from the medical and legal implications, RBD carries the clinical significance of being an early precursor of neurodegenerative process. Hence, early detection and intervention are very important in cases of RBD. Several established RBD scales provide aids for screening, diagnosing, and monitoring treatment progress and response. However, to date, there has been no validated, reliable Turkish scale to diagnose this disorder. The aim of this study was to assess the validity and reliability of a Turkish version of the RBDSQ and to ensure that this tool can be applied in Turkish language.

## 2. Materials and Methods

To create the Turkish adaptation of the RBDSQ, the objectives of this study were explained to RBDSQ developer Stiasny-Kolster, who consented to have the original version translated into Turkish and establish the first RBDSQ-T. Once this was done, an interpreter who was not specialized in medicine translated the Turkish version back into English. The authors of the original RBDSQ were then asked to confirm that the back-translated English document was equivalent to content of the original version. They did so and also gave consent for its use in this study. The instrument was then sent to three experts in Turkey working in different departments of sleep medicine centers, and these individuals agreed that the instrument was applicable for RBD patients in Turkey.

The study was approved by the ethics committee of Hasan Kalyoncu University. It involved three groups: (1) 78 randomly selected healthy controls with no symptoms or history of sleep disorders and no neurological or psychiatric disease, (2) 17 patients previously diagnosed with RBD, and (3) 28 consecutive patients who had been diagnosed with obstructive sleep apnea syndrome (OSAS) but were not treated with continuous positive airway pressure (CPAP) prior to the study. The RBD and OSAS patients were recruited from individuals who were referred to the sleep clinic between January 2013 and August 2014. Obstructive sleep apnea syndrome was diagnosed according to the criteria of American Academy of Sleep Medicine [3], and apnea-hypopnea index greater than 5 per hour was accepted to indicate OSAS. For a patient to be enrolled in the OSAS group, other sleep disorders (e.g., RBD, restless legs syndrome, periodic limb movement disorders, and narcolepsy) were excluded by polysomnography and clinical interview. Polysomnographic evaluation was not performed on the healthy subjects.

All 123 enrolled participants self-rated the RBDSQ-T (preliminary test) during their first visit and were asked to retest them 2.5 months later. The RBDSQ-T comprises 10 items with yes/no questions that address frequency of dreams, dream content, nocturnal movements, injuries to self or bed partner, types of motor behaviors during the night, nocturnal awakenings, sleep disruption, and presence of a neurological disease. Since not all patients have a long-time companion, the bed partner's input was encouraged but not required. Items 1 to 4 address the frequency and content of dreams and their relationship to nocturnal movements and behavior. Item 5 asks about self-injuries and bed partner injuries. Item 6 consists of four subitems that assess nocturnal motor behavior more specifically and questions about nocturnal vocalization, sudden limb movements, complex movements, or items which a bed partner would need to provide answers regarding RBD events. Items 7 and 8 deal with nocturnal awakenings. Item 9 focuses on disturbed sleep in general, and item 10 focuses on the presence of any neurological disorder.

Statistical analyses were performed using SPSS 17.0. General linear model (GLM) univariate analysis of variance was used to compare group results, and the Scheffe multiple comparisons test was used if *F* value in GLM was significant. The diagnostic value of the RBDSQ-T was calculated using area under the curve (AUC) analysis. Test-retest reliability and internal consistency reliability were assessed using Kuder-Richardson (KR) 20 analysis. Descriptive data were presented as means ± standard deviations for continuous variables and as frequencies for categorical variables.

## 3. Results

The 17 RBD patients were five females and 12 males with mean age of 68.6 ± 6.3 years (range, 55–77 years). The 28 untreated OSAS patients were nine females and 19 males with mean age of 50.0 ± 10.8 years (range, 34–77 years), and the average apnea-hypopnea index was 32.5 ± 26.0 per hour. Two RBD patients had Parkinson's disease and three had dementia. The 78 controls were 57 females and 21 males with mean age of 40.0 ± 6.9 years (range, 30–58 years). Of the 123 total participants, 71 (57.7%) were women and 52 (42.3%) were men. The subjects' main demographic features are shown in Table 1.

The Scheffe multiple comparisons test indicated that the mean RBDSQ-T score for the RBD group (9.35 ± 1.58; range, 6–12) was significantly higher than the mean scores for the OSAS group (4.07 ± 2.87; range, 1–12) and healthy controls (2.86 ± 2.15; range, 1–12). The mean score for the RBD group was higher than five, which was defined as the cut-off point for diagnosis of RBD.

When the 123 participants were categorized by sex, the total RBDSQ-T scores for the men were significantly higher than those for the women (*t*(121) = 3.74, *p* = 0.001). The mean score for women was significantly lower than that for men (3.17 ± 2.50 versus 5.21 ± 3.56, resp.; *p* = 0.001).

Within the RBD and OSAS groups, respectively, the mean RBDSQ-T scores for men and women were not significantly different (*t*(15) = 1.69, *p* = 0.110 for RBD; *t*(26) = 0.89, *p* = 0.379 for OSAS). Within the control group, the mean RBDSQ-T score for men was significantly higher than that for women (*t*(76) = 2.84, *p* = 0.006).

Since the distribution of data was normal and variances were homogenous, we performed GLM univariate analysis of variance to compare findings among the RBD group, OSAS group, and healthy group. The results revealed significant differences among these three (*F*(2,120) = 57.04, *p* = 0.001, and *η* ^2^ = 0.49).

**Figure 1:**
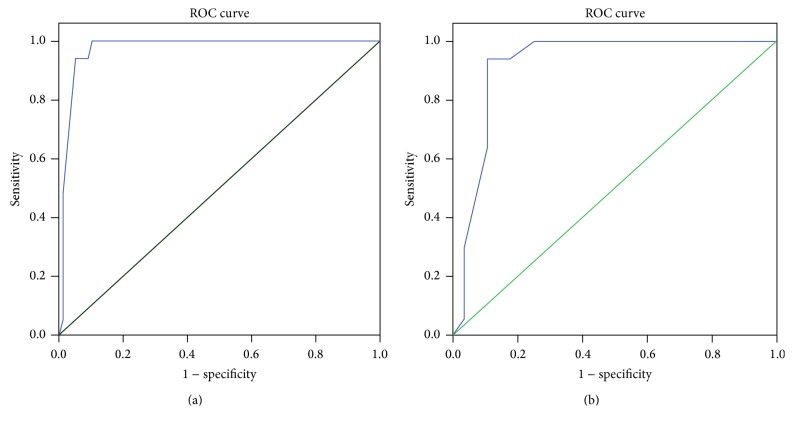
(a) Graph shows the receiver operating characteristic (ROC) curve for the rapid eye movement sleep behavior disorder (RBD) group versus the healthy control group. Area under the curve (AUC) = 0.974 (95% CI: 0.936–1.00; *p* = 0.001). (b) Graph shows the ROC for the RBD group versus the obstructive sleep apnea syndrome (OSAS) group. AUC = 0.921 (95% CI: 0.826–1.00; *p* = 0.001).

Table 2 and Figure 1 demonstrate that, based on a cut-off score of five, the RBDSQ-T was able to discriminate RBD patients from healthy controls with sensitivity of 100% and specificity of 87%. Accordingly, 63% patients were correctly diagnosed using the RBDSQ-T. Similarly, with a cut-off score of five, the RBDSQ-T was able to discriminate RBD patients from OSAS patients with sensitivity of 100% and specificity of 64%.

The diagnostic value of the RBDSQ-T was calculated using the AUC, which was independent of an arbitrary choice of a cut-off point. The resultant values were 0.974 (95% confidence interval (CI): 0.936–1.00) for comparison of the healthy group and the RBD group and 0.921 (95% CI: 0.826–1.00) for comparison of the OSAS group and the RBD group. Single-item analysis revealed the highest specificity for questionnaire items 5, 7, and 10 in comparison with the healthy controls and for items 6.4, 6.3, and 5 in comparison with the OSAS group (Table 3). Regarding test-retest reliability and internal consistency reliability, the test-retest correlation coefficient was 0.95 and the Kuder-Richardson 20 value was 0.82.

## 4. Discussion

The RBDSQ is a concise tool that was developed to assist the clinical evaluation of patients with dream-related behaviors. This self-rating, 13-item questionnaire covers several aspects of sleep- and REM-related behavior, but in some cases bed partners or caregivers information can be needed to cross-check the situation. During our study we did not have that change to do this; all of the participants of research gave their information themselves. And this is one of the limitations of our study, but also other studies have got the same limitations. Our validation study revealed that the Turkish version of the RBDSQ is useful for differentiating RBD patients from OSAS patients and healthy subjects in Turkey. A total score of five was found to be the best cut-off for differentiating RBD patients from healthy controls and OSAS patients.

The mean RBDSQ-T score for the RBD group was 9.3 ± 2.8, which is very close to that noted for the patient group in the original study (9.5 ± 2.8) [4]. The mean score for our RBD group was also higher than corresponding scores in Chinese and Japanese validation studies (8.05 ± 2.46 and 7.5 ± 2.8, resp.) [5, 11]. Even though our study group comprised heterogeneous RBD patients (including two with Parkinson's disease and three with dementia), our RBD patients' mean total score was higher than scores for relevant subgroups in the Chinese study by Wang et al. (8.07 ± 2.71 for symptomatic RBD and 7.95 ± 1.90 for RBD patients with Parkinson's disease) [11]. Those authors concluded that their results reflected differences in symptoms between Asian and European cohorts. Our findings could reflect that the symptoms of RBD in the Turkish population are similar to those that affect European subjects.

Concerning the diagnostic value of the RBDSQ-T, patients with RBD had a significantly higher score for this (9.35 ± 1.58) than the OSAS and control groups (4.07 ± 2.87 and 2.86 ± 2.15, resp.). A cut-off score of five for the RBDSQ-T yielded high sensitivity (100%) but low specificity (64%) for distinguishing RBD patients from those with OSAS. The same cut-off score yielded high sensitivity and specificity (100% and 87%, resp.) for discriminating RBD patients from healthy subjects. A Japanese validation study of the RBDSQ-J [5] showed that a cut-off score of 4.5 yielded very high specificity for differentiating RBD patients from OSAS patients who were under continuous positive airway pressure (CPAP) treatment. The OSAS patients in our study had not been treated, and, in such patients, physical movements during sleep which are associated with abnormal respiratory events can be difficult to differentiate from movements of RBD. The difference in specificity between the Japanese results and ours may be explained by the fact that the Japanese OSAS group had been treated [5]. A Korean validation study of the RBDSQ [12] revealed that a cut-off score of 6.5 yielded high specificity (93.4%) for discriminating RBD patients from untreated OSAS patients. In that investigation, the mean apnea-hypopnea index of patients with idiopathic RBD was 14.8, and this might explain the higher cut-off value and higher specificity compared to our study, in which the RBD patients' apnea-hypopnea index was below 5.

The mean age of our 123 total patients was 46.2 ± 12.6 years. The average age of the RBD patients was 68.6 ± 6.3 years (range, 55–77 years), that of the healthy group was 40.0 ± 6.9 years (range, 30–58 years), and that of the OSAS patients was 50.0 ± 10.8 years (range, 34–77 years). The average ages of groups in the noted Japanese study by Miyamoto et al. also differed [5]: the mean age of the RBD patients was 66.4 ± 6.9 years, that of the healthy group was 64.6 ± 8.8 years, and that of the OSAS patients was 63.1 ± 7.0 years. In the original questionnaire [4], the average ages of the healthy group and RBD group were 50.8 ± 15.5 years and 53.7 ± 15.8 years, respectively. The mean age of our RBD group was similar to the means reported in other studies.

The questionnaire items with most powerful predictive ability were item 8 (100% predictive ability), item 5 (96%), item 6.3 (92.3%), items 2 and 3 (92%), and items 6.1 and 6.2 (87.5%). Because items 2 and 3 address the frequency and content of dreams and their relationship to nocturnal movements and behavior, the answers to these are indicators of RBD. Items 5, 6.1, and 6.2 are related to limb movements and are, therefore, also indicators of RBD. Items 5, 6.1, 6.2, and 6.3 must be answered by bed partners, and we were unable to contact bed partners for each of our participants. Item 8 was a self-report and was difficult to define without bed partners' answers. In the Japanese study [5], Miyamoto et al. found that questionnaire items 1, 2, 5, and 6.1 were the most powerful items.

There are a number of existing scales like Mayo Sleep Questionnaire, RBDQ-Hong Kong (RBDQ-HK), and RBD Single-Question Screen (RBD1Q) for screening and monitoring RBD features [8, 13, 14]. Each of these questionnaires has strong and weak properties. In our study, we prefer RBDSQ because it is easy to administer and can be completed by patients themselves within some points encouraged by bed partners or caregivers; usage of this questionnaire as a screening method demonstrated validity when compared to OSA patients and healthy controls [5]. The weak points of RBDSQ are as follows: not used in monitoring purposes and needs for investigation in differentiating RBD from parasomnia and epilepsy patients [15]. Mayo Sleep Questionnaire is also an easy to administer tool that was completed by bed partners or caregivers [16]. It has questions that might be possible to differentiate RBD from OSA patients. But its application to only patients with cognitive impairments and the fact that there is no information about differentiation from other sleep disorders make this questionnaire limited. Also information taking only bed partners creates questions about self-reports in community usage as a screening method. RBDQ-HK [8] is a more detailed questionnaire for both screening and monitoring RBD and is a very strong questionnaire. Its validation was made in a large scale of patients (RBD, RBD with comorbid with narcolepsy/neurodegenerative disorders, other sleep disorders, and psychiatric patients) and healthy subjects. It is completed by patients and/or bed partners. Its weak point is that it has low sensitivity in differentiating RBD from non-REM parasomnia. RBDQ1 was very sensitive (93.8%), specific (87.2), and easily applicable single-question questionnaire answered by patients encouraged by bed partners or caregivers [14]. But taking RBD patients with a severe symptom from sleep clinic into the validation study and not evaluating non-REM parasomnia patients weaken this questionnaire.

Cronbach's alpha value for the original questionnaire was 0.088 [4], and the corresponding value in the noted Japanese study was 0.086 [5]; Nomura et al. administered the RBDSQ to patients with Parkinson's disease and found Cronbach's alpha to be 0.073 [17]. In our study, because the scale scoring was derived from yes/no questions (i.e., dichotomous scoring), it was considered more appropriate to perform internal consistency reliability analysis using the KR 20 formula instead of Cronbach's alpha. We calculated an internal consistency coefficient of 0.082, which shows that the scale has good internal consistency and that the measuring instrument is reliable.

In conclusion, our findings indicate that the RBDSQ-T is a valid and reliable tool. Comparison of the preliminary test and retest data revealed that the Turkish version of the scale is valid and reliable. RBDSQ-T is a screening questionnaire for the likelihood of harbouring a diagnosis of RBD patients. At that point the questionnaire is only a screening tool and for further confirmatory sleep assessment performing video-polysomnography (v-PSG) will be needed. Nonetheless, this screening questionnaire may help to prioritize the assessment of RBD. For legal perspective, RBD is one of several disorders that can manifest as violent sleep- and dream-related behaviors with forensic and legal implications. In the light of this information, our findings are also important with respect to being able to identify RBD patients who are unknowingly physically violent with their bed partners while sleeping and who may be prosecuted within the justice system for such actions. There is a major loophole in the Turkish judicial system with respect to identifying these patients, and use of the RBDSQ-T could help to determine competence to stand trial in these cases. Apart from the medical and legal implications, RBD carries the clinical significance of being an early precursor of neurodegenerative process. Therefore, early detection and intervention of RBD are the most important steps in this process [13]. Our study also indicates that this scale is a valid and reliable instrument for identifying RBD patients in Turkish language. The availability of a simple and valid screening tool is necessary for both clinical and research purposes; RBDSQ-T could be a significant contribution for researchers in the fields of forensic and criminal psychology, specifically within the judicial system and forensic sciences, as well as for sleep researchers, clinical psychologists, and clinicians in the fields of neurology and sleep.

## Figures and Tables

**Table 1:** Demographic characteristics of the participants.

	RBD group (*n* = 17)	OSAS group (*n* = 28)	Healthy controls (*n* = 78)	Total
Males	12 (70.6%)	19 (67.9%)	21 (26.9%)	52 (42.3%)
Females	5 (29.4%)	9 (32.1%)	57 (73.1%)	71 (57.7%)
Age				
30–45 years	—	10 (35.7%)	—	74 (60.2%)
46–60 years	2 (11.8%)	15 (53.7%)	14 (17.9%)	31 (25.2%)
**>**60	15 (88.2%)	3 (10.7%)	64 (82.1%)	18 (14.6%)

OSAS: obstructive sleep apnea syndrome; RBD: rapid eye movement (REM) sleep behavior disorder.

**Table 2:** Stratified analysis of the rapid eye movement sleep behavior disorders screening questionnaire-Turkish version (RBDSQ-T) results between groups.

Compared with RBD group	Cut-off value	Sensitivity %	Specificity %	Positive predictive value %	Negative predictive value %	Likelihood ratio	AUC (95% CI)	*p* value
Healthy controls	5	100	87	63	100	53.67	0.97 (0.93–1.00)	**0.001**
OSAS group	5	100	64	63	100	24.07	0.92 (0.82–1.00)	**0.001**

AUC: receiver operating characteristic area under curve; RBD: rapid eye movement (REM) sleep behavior disorder; OSAS: obstructive sleep apnea syndrome.

**Table 3:** Sensitivity and specificity of the Turkish version of the rapid eye movement sleep behavior disorder questionnaire items.

RBDSQ-T item	Sensitivity (%)		Specificity (%)		Positive predictive value (%)		Negative predictive value (%)	
	Controls	OSAS patients	Controls	OSAS patients	Controls	OSAS patients	Controls	OSAS patients
(1) I sometimes have very vivid dreams.	100	100	21.79	25	21.79	44.74	100	100
(2) My dreams frequently have an aggressive or action-packed content.	88.24	88.24	87.18	82.14	60	75	97.14	92
(3) The dream contents mostly match my nocturnal behavior.	88.24	88.24	83.33	82.14	53.57	75	97.01	92
(4) I know that my arms or legs move when I sleep.	29.41	29.41	78.21	46.43	22.73	25	83.56	52
(5) It thereby happened that I (almost) hurt my bed partner or myself.	94.12	94.12	97.44	85.71	88.89	80	98.7	96
(6) I have or had the following phenomena during my dreams:								
(6.1) Speaking, shouting, swearing, laughing loudly.	82.35	82.35	67.95	75	35.9	66.67	94.64	87.5
(6.2) Sudden limb movements, “fights.”	82.35	82.35	88.46	75	60.87	66.67	95.83	87.5
(6.3) Gestures, complex movements, that are useless during sleep, for example, to wave, to salute, to frighten mosquitoes, falls off the bed.	88.24	88.24	87.18	85.71	60	78.95	97.14	92.31
(6.4) Things that fell down around the bed, for example, bedside lamp, book, glasses…and so forth.	47.06	47.06	92.31	89.29	57.14	72.73	88.89	73.53
(7) It happens that my movements awake me.	64.71	64.71	94.87	78.57	73.33	64.71	92.5	78.57
(8) After awakening I mostly remember the content of my dreams well.	100	100	87.18	35.71	62.96	48.57	100	100
(9) My sleep is frequently disturbed.	41.18	41.18	34.62	64.29	12.07	41.18	72.97	64.29
(10) I have/had a disease of the nervous system (stroke, head trauma, parkinsonism, RLS, narcolepsy, depression, epilepsy, inflammatory disease of the brain), which?	29.41	29.41	93.59	67.86	50	35.71	85.88	61.29

RBDSQ-T: RBD screening questionnaire-Turkish version; RBD: rapid eye movement (REM) sleep behavior disorder; OSAS: obstructive sleep apnea syndrome.
